# Pharmacokinetics, bioavailability, and excretion of ponazuril in piglets

**DOI:** 10.3389/fvets.2022.1054417

**Published:** 2022-12-07

**Authors:** Wenxiang Wang, Yuqiao Ma, Yunxiao Zhang, Jingjing Nie, Daxing Hu, Weicong Yang, Yue Shen, Xinglong Cui, Huanzhong Ding, Li Li, Xianhui Huang

**Affiliations:** ^1^Guangdong Provincial Key Laboratory of Veterinary Pharmaceutics Development and Safety Evaluation, College of Veterinary Medicine, South China Agricultural University, Guangzhou, China; ^2^College of Animal Science, South China Agricultural University, Guangzhou, China

**Keywords:** piglets, excretion, bioavailability, ponazuril, nonlinear pharmacokinetics, NLME

## Abstract

Ponazuril is a triazine anticoccidial drug which is the main metabolite of toltrazuril in animals, it has excellent activity against many protozoa, including *Cystoisospora suis*, and has broad application prospects in the control of swine coccidiosis. To evaluate the pharmacokinetic and excretion characteristics of ponazuril, 12 healthy piglets aged 10–14 days were divided into 2 groups for pharmacokinetic studies, which were given 20 mg/kg body weight ponazuril orally and intravenously, respectively. And 6 other piglets were housed individually in metabolic cages and given the same oral dose of ponazuril. After administration, the concentration of ponazuril in plasma, fecal, and urine samples collected was determined using high-performance liquid chromatography (HPLC). The plasma concentration profiles of ponazuril obtained after intravenous and oral administration were analyzed simultaneously by the nonlinear mixed-effects (NLME) model. Following the results, the pharmacokinetics of ponazuril exhibited a Michaelis-Menten elimination with Michaelis-Menten constant K_m_ and maximum metabolic rate V_m_ of 10.8 μg/mL and 0.083 mg/kg/h. The apparent volume of distribution was calculated to be 735 mL/kg, and the final estimated oral bioavailability was 81%. Besides, cumulatively 86.42 ± 2.96% of ponazuril was recovered from feces and 0.31% ± 0.08% from urine during 0–1,020 h after oral administration. These findings indicated a good oral absorption of ponazuril in piglets with nonlinear disposition and slow excretion largely *via* feces, implying sustained drug concentration *in vivo* and long-lasting anticoccidial effects.

## Introduction

*Cystoisospora suis* (*C. suis*) is one of the most important pathogens causing swine coccidiosis (1), which often leads to serious damage to the epithelial mucosa of the jejunum and ileum of piglets, characterized by non-hemorrhagic diarrhea, resulting in reduced growth performance and even death of piglets (2, 3), thus causing significant economic losses to the pig farming industry. However, various drugs are also being used for the control of coccidia in animals, Noack et al. have summarized in detail about the anticoccidial drugs of the livestock industry, which include two main categories of polyether antibiotics or ionophores (such as monensin, salinomycin, maduramicin, etc.) and synthetic compounds (such as sulfonamides, amprolium, diclazuril, etc.) (4). Toltrazuril, a triazine antiprotozoal drug, has been approved by the European Medicines Agency (EMA) for use in coccidiosis in chickens and pigs (5–7). It acts on the entire intracellular developmental stage of the protozoa (8, 9) and exhibits excellent pharmacological activity, which has been confirmed by relevant *in vitro* and *in vivo* studies (5, 10–13).

Ponazuril is a triazine drug with a molecular weight of 457 and partition coefficients (LogP) of 3.1 (14), also known as toltrazuril sulfone, is the major metabolite of toltrazuril in animals (5, 6), which has similar properties to toltrazuril. Ponazuril is currently approved in veterinary medicine only for the treatment of equine protozoal myeloencephalitis (15). Moreover, it also has excellent activity against other parasites such as coccidia, *Neospora caninum*, and *Toxoplasma gondii* (5, 16–19), demonstrating a broad development prospect in the future.

Certainly, the absorption, distribution, metabolism, and excretion characteristics of drugs in animals are necessary studies before they can be applied. The pharmacokinetic studies of toltrazuril in different animals have been gradually revealed, characterized by good oral absorption, long residence time in the body, and significant interspecies differences (5, 20–22). Although toltrazuril is mostly metabolized to ponazuril and excreted through feces, which is similar in chickens, pigs, and rats (5, 6), the kinetics of ponazuril itself *in vivo* need to be further clarified.

Currently, the pharmacokinetics of ponazuril has been reported in a variety of animals including cattle (23), goats (24), camels (25), cats (26), horses (27), turtles (28), and weaned pigs (29). But no relevant studies targeting ponazuril in piglets aged 0–4 weeks, which is the optimal administration time to control coccidiosis, have been published thus far. To further characterize and validate the absorption, elimination, and excretion of ponazuril at different animal and day ages, particularly, we used 2-week-old piglets for pharmacokinetics and excretion studies of ponazuril and set up an intravenous administration to assess its oral bioavailability.

## Materials and methods

### Chemicals and reagents

Ponazuril reference standard (100%), ponazuril suspension (5%), and ponazuril injection (5%) were provided by Hubei Longxiang Pharmaceutical tech. co., ltd. (Huanggang, China). Acetonitrile and methanol [high-performance liquid chromatography (HPLC) grade] were purchased from ANPEL Laboratory technologies (Shanghai) Inc. (Shanghai, China). Water was purified using a water purification system from Kangning Tech (Chengdu, China). Hydrophilic lipophilic balance (HLB) solid phase extraction (SPE) column was purchased from Waters Corporation (Milford, MA, USA). Other chemicals used were of analytical grade and purchased from Damao Chemical Reagent Factory (Tianjin, China).

### Animals and feeding

Ethical approval for all experiments in pigs was obtained from the Animal Ethics Committee of South China Agriculture University. A total of 18 healthy piglets (Yorkshire × Laiwu) weighing 3.36 ± 0.32 kg, 10–14 days aged, were used in this study. The animals were housed in an environment with suitable temperature and humidity, given standard commercial suckling pig feeds 3 times a day, and supplemented by artificial milk. Water was provided *ad libitum* during all experiments.

### Pharmacokinetic study

Pigs were randomly divided into 2 groups (*n* = 6 each), Group A received a single oral administration of 20 mg/kg body weight (bw) of ponazuril suspension, and blood samples were collected at 0.5, 1, 6, 12, 24, 36, 48, 72, 96, 144, 240, 360, 480, 720, and 960 h after administration. Group B received ponazuril intravenously at the same dose as Group A, and blood samples were collected at 0.17, 1, 6, 12, 24, 48, 72, 96, 144, 192, 240, 360, 480, 720, and 960 h after administration. The doses were selected concerning the recommended dose of toltrazuril suspension in piglets (7, 30). All blood samples (~2 mL) were collected from the anterior vena cava and transferred to heparinized polypropylene centrifuge tubes, centrifuged at 1,250 × *g* for 10 min, and the plasma was collected and stored at −20°C until analysis.

### Excretion study

Six healthy piglets were housed individually in metabolic cages and given a single oral administration of 20 mg/kg bw of ponazuril suspension. Urine and fecal samples were collected at intervals of 0–12, 12–24, 24–36, 36–60, and 60–84 h, and every 24 h thereafter until 1,020 h when we could not accurately determine ponazuril in the samples. The urine and feces samples were stored at −20°C until analysis.

### Sample pretreatment

The selectivity was investigated by analyzing and comparing blank plasma, urine, and feces from the animals before administration with the corresponding spiked matrices and the samples collected after dosing. And the samples were extracted by adding appropriate concentrations of ponazuril to blank plasma, fecal, and urine samples as a way to evaluate the linearity as well as the recovery accuracy and precision of ponazuril.

#### Plasma procedure

The plasma samples were thawed at room temperature and vortexed. 1 mL of acetonitrile was added to 500 μL of plasma sample in a 2 mL centrifuge tube, vortexed for 1 min, and centrifuged at 5,590 × *g* for 10 min at 4°C. The supernatant of the mixture was evaporated under a nitrogen stream at 45°C. The residue was re-dissolved with 1 mL of acetonitrile/water (46/54, v/v), and filtered through a 0.22 μm filter for HPLC analysis.

#### Feces procedure

The fecal samples were thawed at room temperature and vortexed. 1 g of each fecal sample was weighed into a 50 mL centrifuge tube and 10 mL of acetonitrile was added. The mixture was vortexed for 3 min, shaken for 20 min, and centrifuged at 6,700 × *g* for 15 min at 4°C, 2 mL of supernatant was transferred to a glass tube, and 7 mL of water was added to obtain the reserve solution. An HLB SPE column (60 mg/3 mL) was activated successively with 3 mL methanol and water, and the reserve solution was all drawn into the extraction column, then rinsed successively with 3 mL water and acetonitrile/water (3/7, V/V), and eluted with 3 mL acetonitrile. The eluate was evaporated under a nitrogen stream at 45°C. Finally, the residue was re-dissolved with 1 mL of methanol/water (1/1, v/v), and filtered through a 0.22 μm filter for HPLC analysis.

#### Urine procedure

The urine samples were thawed at room temperature and vortexed. 1.0 mL of each urine sample was aspirated into a 15 mL centrifuge tube and 2 mL of 0.2% acetic acid in acetonitrile was added. The mixture was vortexed for 3 min and centrifuged at 5,590 × *g* for 10 min at 4°C. 3 mL dichloromethane was added into the supernatant before the mixture was vortexed for 3 min and centrifuged (2,740 × *g*, 4°C) again. Then the lower phase liquid was evaporated in a new glass tube under a nitrogen stream at 45°C, and the residue was re-dissolved with 1 mL of methanol/water (1/1, v/v), and filtered through a 0.22 μm filter for HPLC analysis.

### HPLC-UV instrument and analytical conditions

The ponazuril in all biological samples was analyzed by an HPLC system equipped with an LC-20AT pump, an SPD-20A UV detector (Shimadzu, Kyoto, Japan), and a Kinetex EVO C18 column (250 × 4.6 mm, 5 μm) (Phenomenex, USA). The mobile phases used were potassium dihydrogen phosphate solution (0.680 g of potassium dihydrogen phosphate was dissolved in 900 mL of water. The pH value was adjusted to 5.00±0.05 with potassium hydroxide solution and made to a constant volume of 1 L) (A), acetonitrile (B), and 0.1% formic acid in water (C). The plasma, urine, and fecal samples were determined using an isocratic mobile phase of C/B (54/46), A/B (56/44), and A/B (55/45), respectively, with follow rate of 1 mL/min and detection wavelength at 255 nm.

### Pharmacokinetic analysis

**Figure 1:**
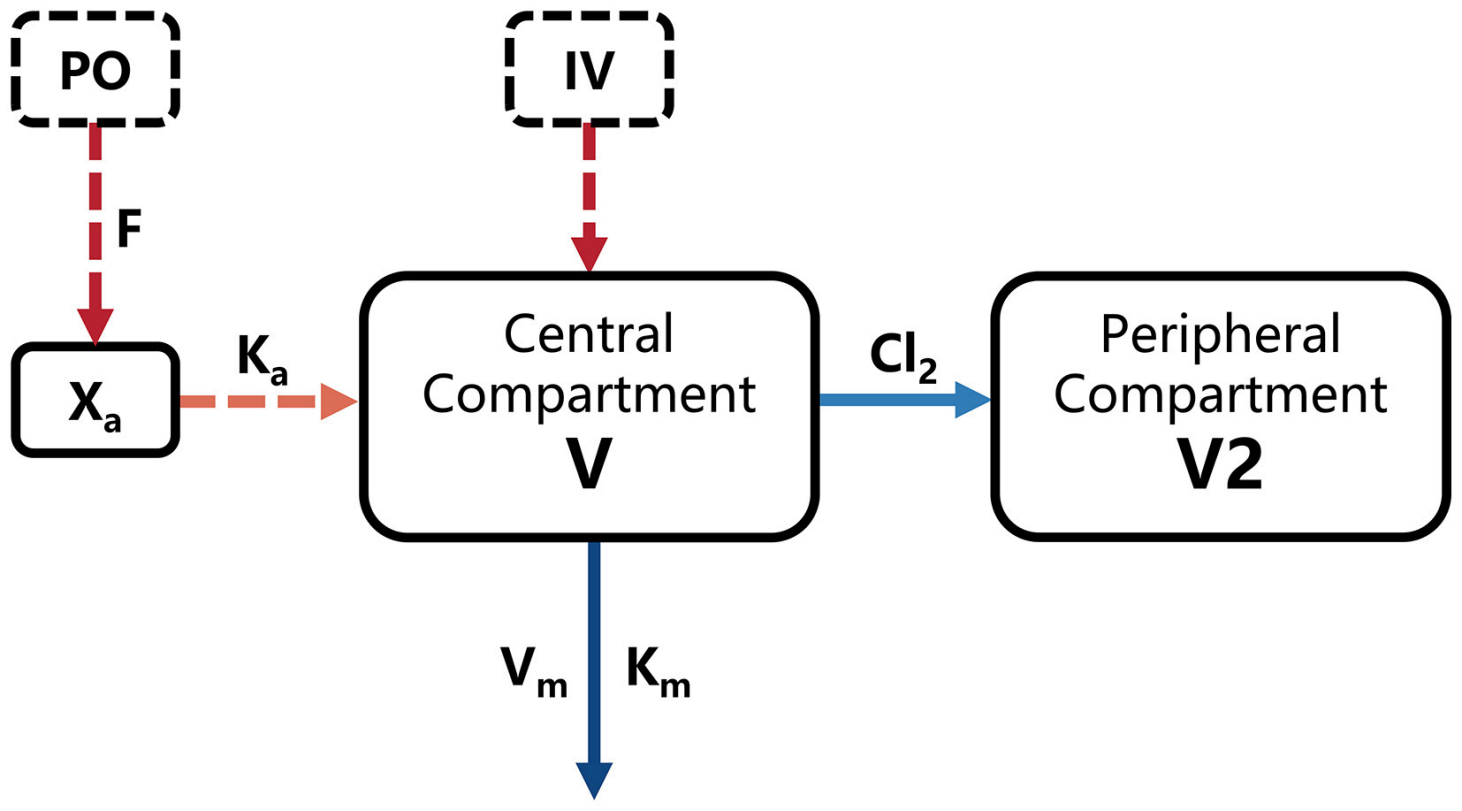
Final model structure of ponazuril pharmacokinetics following oral and intravenous dosing in healthy piglets. V, central volume of distribution; V2, peripheral volume of distribution; K_m_, Michaelis-Menten constant; V_m_, maximum metabolic rate; Cl_2_, clearance from central to peripheral compartment; K_a_, absorption rate constant; F, absolute oral bioavailability; X_a_, absorbed dose.

Simultaneous analysis of intravenous (IV) and oral (PO) concentration-time data was based on the nonlinear mixed-effects (NLME) model in Phoenix 8.1 (Cetera, USA). A two-compartment model including a Michaelis-Menten elimination (Figure 1) was built to fit the pharmacokinetic process of ponazuril in piglets, and the population-level parameters (fixed effects) were estimated using Quasi-Random Parametric Expectation Maximization (QRPEM) algorithm with a multiplicative + additive residual error model. In the modeling process, no covariates were added and the random effects of between-subject variability (BSV) on model parameters were evaluated for the structural parameters V, V_m_, and Cl_2_, with an exponential model as follows:


(1) $$\begin{array}{l} Pi=\theta \cdot exp\;\left(\eta i\right) \end{array}$$

where P_i_ is the parameter estimate for ith individual, θ is the typical value of the parameter in the population. η_i_ is a random variable of individual i with mean of zero and variance of ω^2^.

The selection and optimization of the model structure were guided by the Akaike's information criterion (AIC), Bayesian information criterion (BIC), −2 log-likelihood criterion (-2LL), and graphical analysis of observed vs. model-predicted concentrations at the population and individual levels. Besides, the situation of conditional weighted residuals (CWRES) over the population predicted concentrations and vs. time was evaluated. For the final model structure, a bootstrap analysis was performed by resampling 200 times from the random selection to verify the stability and accuracy of various model parameter estimates at 95% confidence intervals (CI). And a visual predictive check (VPC) was used to evaluate the ability of the model to predict variability in observed data, this consisted of the simulation of 2,000 hypothetical samples with the final model and the fit between the simulated and observed quantiles.

When the pharmacokinetics of ponazuril had to be analyzed with the Michaelis-Menten model, we also used the linear trapezoidal method from noncompartmental analysis (NCA) to calculate the area under the concentration-time curve (AUC) from 0 to last measurable concentration, while comparing it with the NLME model estimates to assess the bias of the results.

## Results

### Chromatographic characteristics of ponazuril

**Figure 2:**
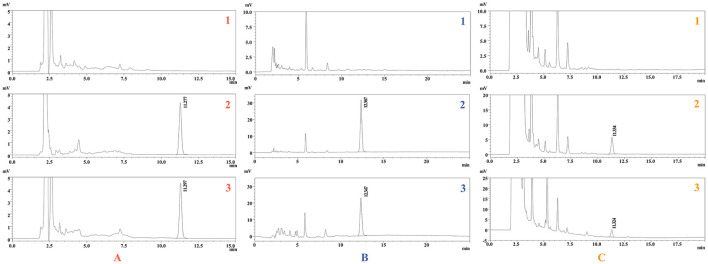
Representative chromatogram of ponazuril. Blank plasma **(A1)**, feces **(B1)**, and urine **(C1)**; Spiked plasma (2 μg/mL) **(A2)**, feces (25 μg/g) **(B2)**, and urine (1 μg/mL) **(C2)**; Plasma **(A3)**, feces **(B3)**, and urine **(C3)** samples collected after oral administration of ponazuril (20 mg/kg bw).

As shown in Figure 2, the target peak was well resolved, with satisfactory shapes, and the retention times of ponazuril in the plasma, fecal, and urine matrices were approximately 11.2, 12.3, and 11.3 min, respectively.

### Method validation

The standard curves for ponazuril were linear (r>0.99) in the range of 0.1–20 μg/mL for plasma, 0.25–100 μg/g for feces, and 0.05–5 μg/mL for urine, the respective regression equation, as well as the limits of detection (LOD) and limits of quantification (LOQ) for each biological sample are summarized in Table 1.

**Table 1:** Calibration curve and LOD&LOQ of ponazuril.

**Matrixes**	**LOD and LOQ**	**Linear range**	**Regression equation**	**Determination coefficient (r^2^)**
Plasma	0.02 and 0.1 μg/mL	0.1–20 μg/mL	C = 3.7432 × 10^−5^A−0.0233	0.9994
Feces	0.1 and 0.25 μg/g	0.25–100 μg/g	C = 1.2673 × 10^−5^A−0.0333	0.9988
Urine	0.02 and 0.05 μg/mL	0.05–5 μg/mL	C = 1.2843 × 10^−5^A−0.0054	0.9996

A, peak area of ponazuril; C, concentrations of ponazuril in biological samples.

The results of the methodological validation are listed in Table 2. The mean recoveries in plasma, feces, and urine ranged from 97.3 to 102.0%, 89.6 to 95.9%, and 93.5 to 99.2%, with intraday variation ranging from 0.43 to 4.27%, 1.76 to 3.17%, and 0.97 to 7.62%, and interday variation ranged from 0.65 to 3.58%, 2.40 to 3.63%, and 1.16 to 6.43%, respectively.

**Table 2:** Recovery and precision values for the determination of ponazuril.

**Concentrations of ponazuril**		**Average intraday recovery (%)**			**Coefficient of intraday variation (%)**			**Average interday recovery ($\bar{\text{X}}$ ±SD) (%)**	**Coefficient of interday variation (%)**
		**1**	**2**	**3**	**1**	**2**	**3**		
Plasma (μg/mL)	0.1	98.7	102.0	97.3	2.30	4.27	2.41	99.4 ± 3.56	3.58
	2	99.8	100.7	98.3	1.01	0.43	0.74	99.6 ± 1.25	1.25
	20	99.7	100.0	99.0	0.43	0.71	0.44	99.6 ± 0.65	0.65
Feces (μg/g)	0.25	95.9	89.6	90.6	1.76	2.30	2.00	92.0 ± 3.34	3.63
	25	92.0	93.6	94.0	2.29	2.46	2.34	93.2 ± 2.24	2.40
	100	93.9	95.2	94.5	2.77	2.97	3.17	94.5 ± 2.67	2.82
Urine (μg/mL)	0.05	93.5	97.6	93.5	7.62	5.95	5.78	94.9 ± 6.10	6.43
	1	98.3	98.8	98.8	1.34	0.97	1.29	98.6 ± 1.15	1.16
	5	99.2	97.4	98.9	6.15	6.09	5.87	98.5 ± 5.64	5.73

$\bar{\text{X}}$ ± SD, mean ± standard deviation.

### NLME parameter estimates and model evaluation

**Figure 3:**
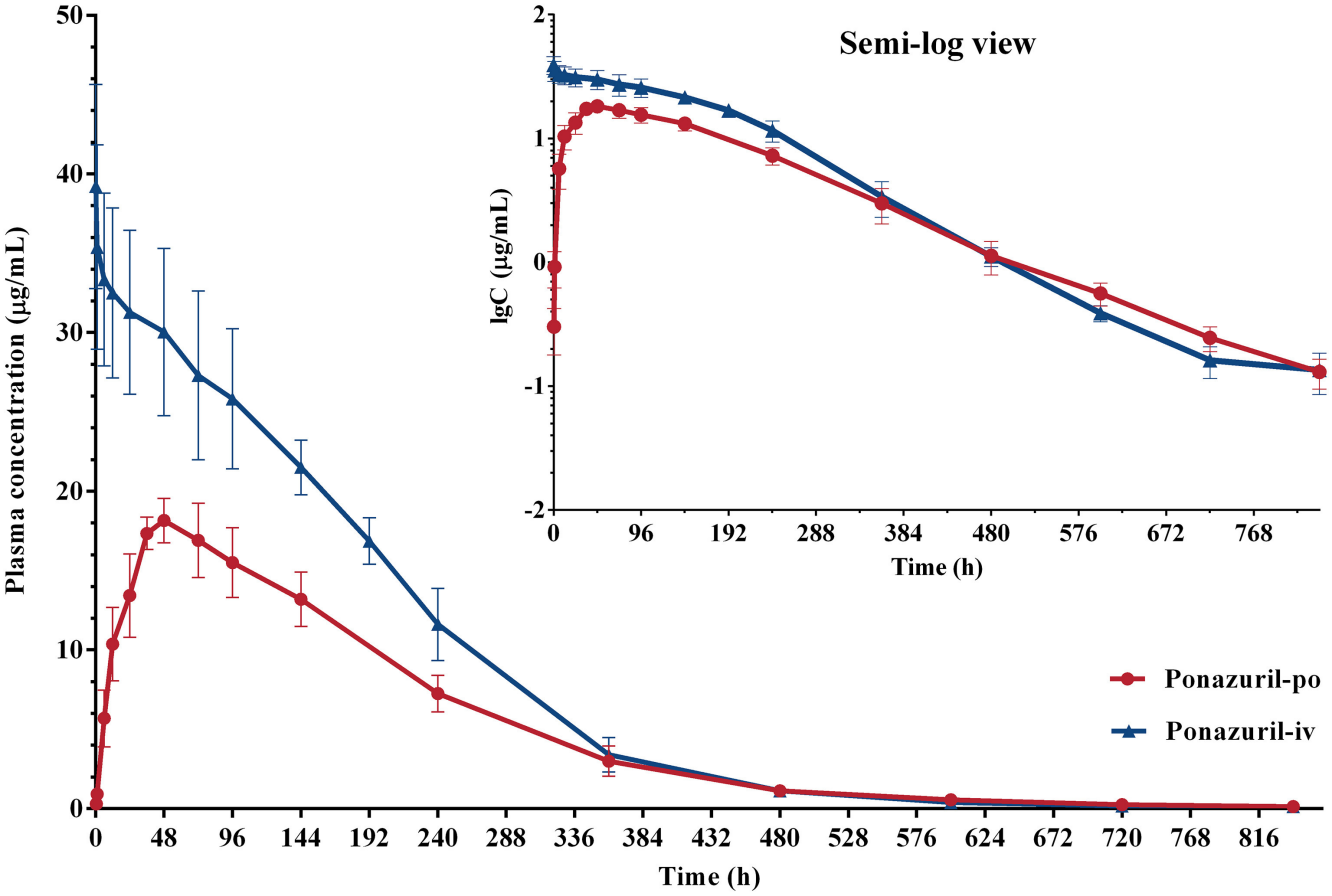
Plasma concentration-time curves of ponazuril (20 mg/kg bw) in piglets after oral administration and intravenous injection. Data represent mean ± SD values for 6 pigs (linear scale; inset: semi-logarithmic scale).

The concentration-time data of ponazuril in piglets after oral and intravenous administration is summarized in Supplementary Tables 1, 2. The blood concentration-time curve is shown in Figure 3.

**Figure 4:**
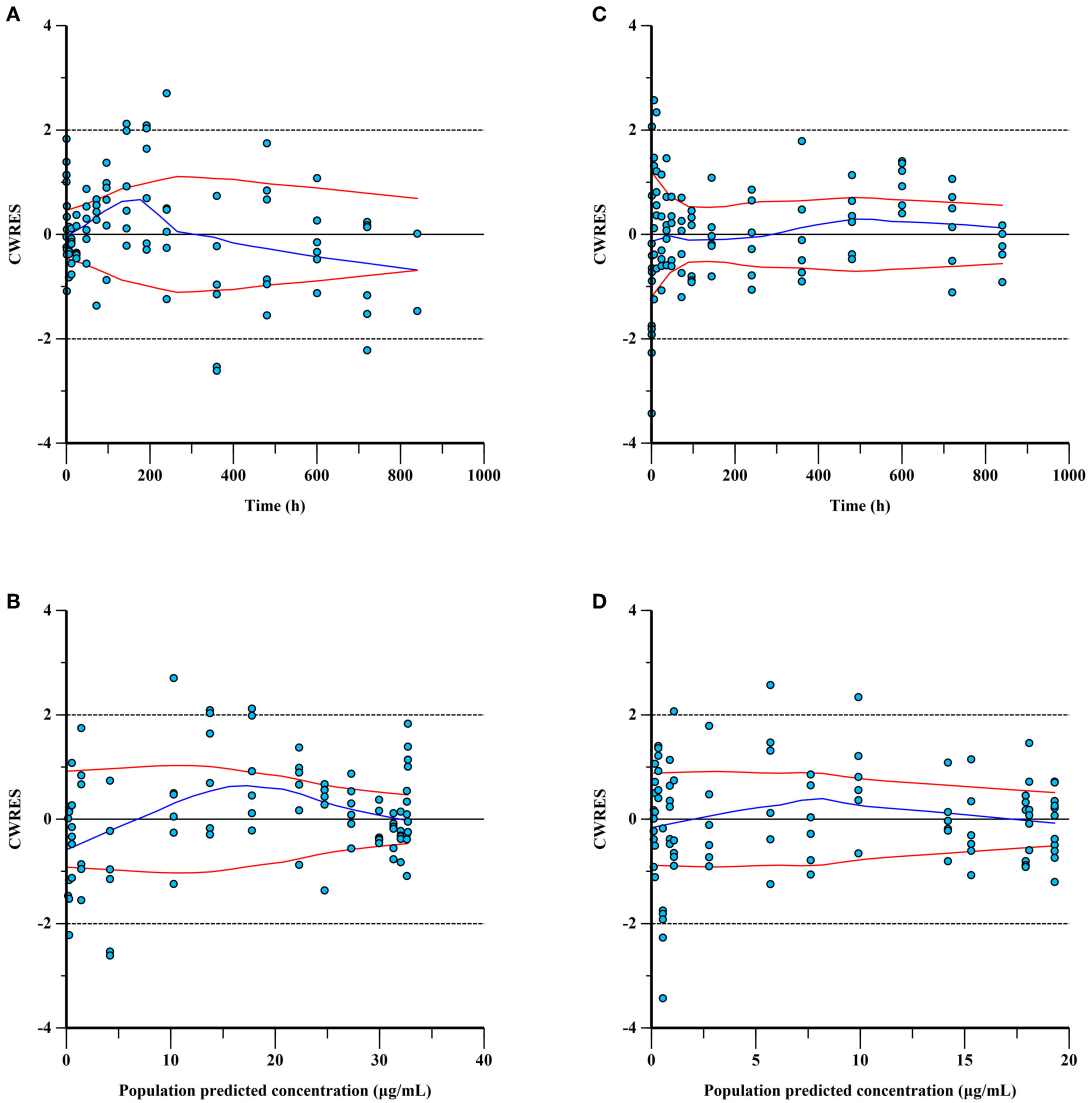
Conditional weighted residuals (CWRES) for ponazuril plasma concentrations: CWRES of IV data versus time **(A)** and population predicted concentrations **(B)**; CWRES of PO data vs. time **(C)** and population predicted concentrations **(D)**. CWER should be mainly distributed between y = 2 and y = −2 to prove the goodness of fit.

**Figure 5:**
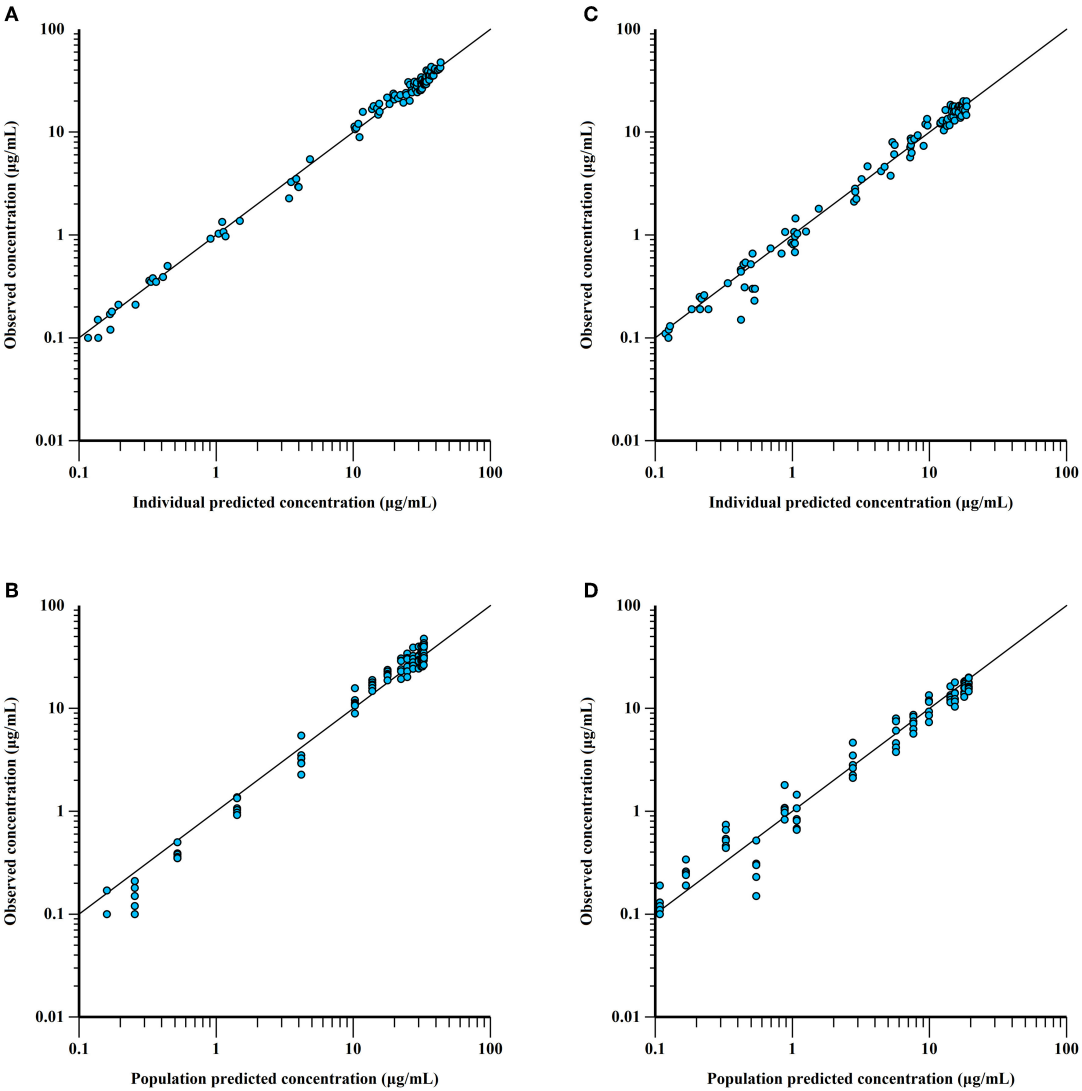
Observed vs. predicted ponazuril plasma concentrations (logarithmic scale). IV: observed vs. individual **(A)** and population **(B)** predicted concentrations; PO: observed vs. individual **(C)** and population **(D)** predicted concentrations. The proximity of the blue points to the uniform line y = x reflects the goodness of fit.

**Figure 6:**
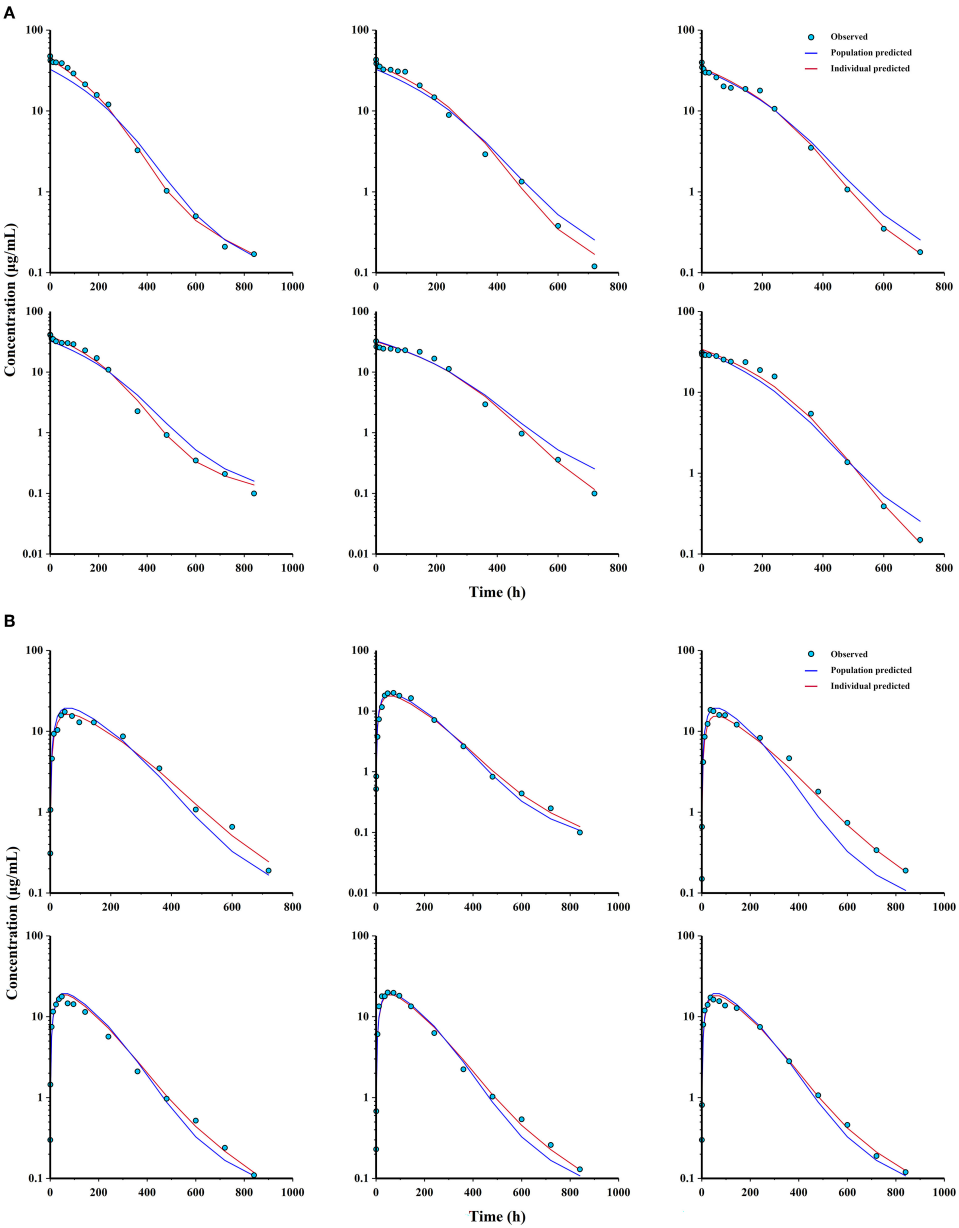
Observations, population and individual predictions vs. time profiles for ponazuril (semi-logarithmic scale). **(A)** individuals after IV administration (*n* = 6); **(B)** individuals after PO administration (*n* = 6).

Combined with the observation of concentration-time data and the smaller values of −2LL, AIC, and BIC, a two-compartment model with Michaelis-Menten elimination for the IV and PO route was selected to best fit the pharmacokinetics of ponazuril in plasma. As seen in Figures 4–6, visual inspection of the goodness-of-fit plots, which consist of the distribution of CWRES and the bias between model population and individual predictions and observed concentrations of ponazuril, all showed acceptable goodness-of-fit for the final model of ponazuril. Table 3 summarized the parameter estimates of the final model, the population bioavailability was calculated as 81%, with an estimated absorption rate constant of 0.041 1/h. The volume of distribution was 611 and 124 mL/kg for the central and peripheral compartments, respectively. And the values of Michaelis Menten kinetic parameters K_m_ and V_m_ were estimated as 10.8 μg/mL and 0.083 mg/kg/h.

**Table 3:** Estimated pharmacokinetic parameters of ponazuril after oral and intravenous administration and bootstrap precision diagnostic results.

**Parameter**	**QRPEM**			**Bootstrap analysis**			
	**Estimate**	**CV (%)**	**BSV (%)**	**2.5^th^ percentile**	**Median estimate**	**97.5^th^ percentile**	**CV (%)**
V (mL/kg)	611	22.96	2.34	552	610	695	6.30
V2 (mL/kg)	124	64.23	/	81	117	212	63.99
K_m_ (μg/mL)	10.8	33.15	/	6.9	10.6	17.7	26.80
V_m_ (mg/kg/h)	0.083	16.02	0.09	0.064	0.083	0.105	12.90
Cl_2_ (mL/h/kg)	0.31	94.76	44.32	0.13	0.34	0.67	38.29
Ka (1/h)	0.041	11.56	/	0.033	0.041	0.055	13.49
F (%)	81	10.00	/	67	82	87	5.27
AUC (h·μg/mL)
NLME	6,640 ± 419 (4,045 ± 108)						
NCA	6,893 ± 623 (4,116 ± 301)						

CV, coefficient of variation of the estimated parameter; BSV for random effects parameters expressed as CV% (ω^2^) and calculated as $CV\%\left(\omega^{2}\right)=\sqrt{\exp\left(\omega^{2}\right)-1}\times 100\%$; AUCs were estimated separately at individual level by the final NLME model and NCA, and presented as mean ± SD, distinguished by IV (PO) values.

**Figure 7:**
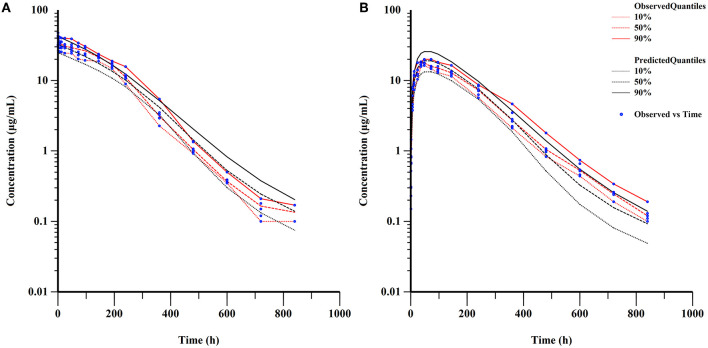
Visual predictive check of final model (semi-logarithmic scale). **(A)** concentrations after IV administration; **(B)** concentrations after PO administration.

Table 3 also provided the median with 95% CI parameter estimates obtained from 200 bootstrap operations, which are highly close to the parameters finally estimated by the QRPEM algorithm, suggesting the accuracy and stability of the model. In addition, the VPC simulation plot (Figure 7) showed that the predicted quantiles profiles follow the same trend as the observed values with a prediction interval of 10–90%, indicating that the final NLME model reliably estimated and predicted the pharmacokinetic parameters of ponazuril.

### Excretion study

**Figure 8:**
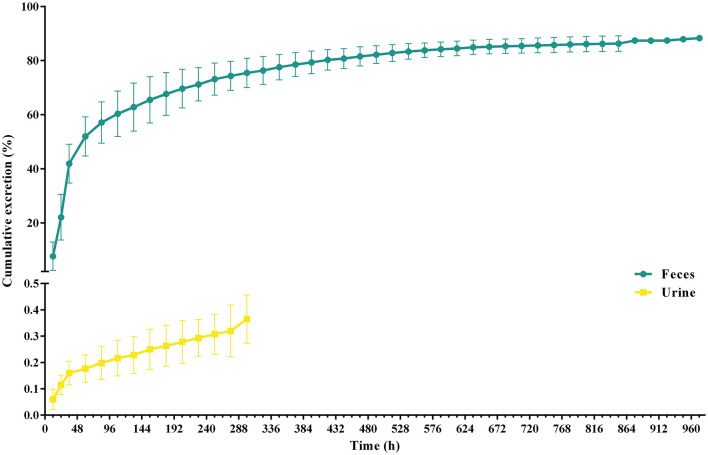
Urinary and fecal cumulative excretion profiles of ponazuril (20 mg/kg bw) after oral administration. Data represent mean ± SD values for 6 pigs.

After a single oral administration, the cumulative excretion curve of ponazuril in the feces and urine of piglets is shown in Figure 8, and detailed excretion data can be found in the Supplementary Tables 3, 4. Cumulatively, 86.42 ± 2.96% of ponazuril was recovered in feces and 0.31 ± 0.08% in urine during the sampling period of 0–1,020 h (42.5 d). The majority of ponazuril was excreted in the feces and urine at around 972 h (40.5 d) after dosing. The results indicated that ponazuril was excreted slowly, mainly in the feces, with a small amount appearing in the urine.

## Discussion

In this study, following intravenous administration, as demonstrated in Figure 3, the plasma concentration of ponazuril exhibited a typical plateau phase of nonlinear disposition in the first 200 h, after which concentrations declined rapidly and elimination slowed again around 600 h. This nonlinear process means that it could not be accurately analyzed by conventional compartmental models, and to obtain kinetic parameters for the absorption and elimination of ponazuril in piglets, particularly bioavailability, V_m_, and K_m_, this required us to analyze intravenous and oral data simultaneously. Considering that all individual pharmacokinetic data of ponazuril were obtained from different piglets, making the application of the NLME model possible. With the evaluation of −2LL, AIC, BIC, and other model diagnostic results, we fitted the pharmacokinetic process of ponazuril well using a two-compartment model including Michaelis Menten elimination. And the absolute oral bioavailability of ponazuril estimated by the final NLME model was approximately 81%, while the bioavailability calculated using the AUC ratio method was about 60%, obviously the former is closer to the actual situation. As mentioned in the literature review, the calculation of bioavailability in terms of AUC is based on the premise that the clearance of a drug *in vivo* is constant, i.e., the AUC is proportional to the total amount of drug reaching the systemic circulation. In contrast, when the drug exhibits Michaelis-Menten elimination, the clearance depends on the concentration, which may lead to a small AUC estimate after extravascular administration (31). Jusko et al. also found that the bioavailability determined by the Michaelis-Menten approach was higher than the values determined by the conventional method of area ratios (32). And for the clearance, when the pharmacokinetic was linear, we derived its value from V_m_/K_m_ as 7.7 mL/h/kg whereas NCA gave 2.9 mL/h/kg from the dose to AUC ratio. The present findings provide evidence that the NCA approach is not absolutely applicable to the pharmacokinetic analysis of ponazuril in pigs. However, similar results had not been reported in any previous literature, which requires further consideration for the *in vivo* disposition of ponazuril in future studies.

Furthermore, the apparent volume of distribution estimated by NLME was 735 mL/kg. In general, animal total body fluid represents 60% of body weight and plasma is 4.5–5% (33), Tollerz reported total body water content represents approximately 72.8% of body weight in piglets (34), and for piglets in this study with an average body weight of around 3.4 kg, the apparent volume of distribution of ponazuril was similar to the total body fluid volume, suggesting a high degree of systemic distribution. Of note, the central distribution volume was much larger than the peripheral distribution volume, revealing that ponazuril is mostly distributed in the plasma as well as in some blood flow-rich tissues and organs in pigs (35), which may be associated with the lipophilicity of ponazuril (LogP = 3.1). For the model fits, the CV% of V2 and Cl_2_ estimated by NLME were high and the bootstrap procedure gave better values, but still >30%. This is an estimation issue, which may be linked to the fact that the data collected was insufficient to support the algorithm for the peripheral compartment parameters, but the results returned by other model diagnostics were all satisfactory, including the fitted individual AUCs that were similar to the values calculated by NCA, verifying that the accuracy of the model fit was reliable.

Nonlinear kinetics occurs when one or more processes of absorption, distribution, metabolism, or excretion are saturable. That is a situation in which the rate or extent of a process cannot increase proportionally to the dose or concentration and approaches an upper limit (36). For ponazuril or toltrazuril, there are no early findings to demonstrate its saturability in physiological processes, but we can speculate from the pharmacokinetic results about the possibility of its non-linear absorption or saturable metabolism. Zec et al. reported that ponazuril in peafowl was absorbed orally with C_max_ of 11.82 μg/mL and 18.42 μg/mL for 20 and 40 mg/kg doses, respectively. But the increase in plasma concentration and AUC were not proportional to a doubling of the dose, indicating potential nonlinear absorption in these birds (37). From the present study in piglets, ponazuril peaked at about 48 h after a single oral dose of 20 mg/kg bw with a mean concentration of 18 μg/mL. In comparison, Zou et al. found that the blood concentration of ponazuril peaked at 5.8 μg/mL at around 42 h after a single oral dose of 5 mg/kg bw to 6 weaned pigs aged 2–3 months (29), and it could be noticed that the degree of absorption did not increase proportionally to the dose. Of course, the gastrointestinal physiology of pigs of various ages is different, such as the pH in the digestive tract will fluctuate before and after weaning (38, 39), and the gastrointestinal emptying time in piglets is shorter than that of growing pigs (40–43), etc. We found no evidence of possible carrier system saturation, plasma protein binding saturation, or saturable first-pass metabolism of ponazuril, mechanisms that are usually associated with nonlinear absorption (44). Another point, both toltrazuril and its metabolites are poorly soluble in water (45), the lipophilicity will cause an upper limit to its solubility in the gastrointestinal environment, which may lead to a misestimation of bioavailability. Pharmaceutical dosage forms should also be considered. Furr et al. demonstrated that the combination of ponazuril with oil increased the serum and cerebrospinal fluid concentrations of ponazuril in horses after oral administration (27). Dirikolu et al. used dimethyl sulfoxide (DMSO) as an oral solvent for ponazuril and performed pharmacokinetic tests in horses, and the results of cross-testing of ponazuril showed that the absolute bioavailability of ponazuril in DMSO was 71%, which was about 3-fold higher compared to the aqueous suspension. And the absorption half-life also increased from 6.16 to 7.91 h (46). The current study assessed an unbiased bioavailability using the NLME model, yet the main shortcoming is the absence of a multi-dose experimental design, which would help to examine the potential non-linear mechanisms of ponazuril more scientifically.

More importantly, thanks to the collected intravenous data, we clearly observed the saturation kinetic process of ponazuril. Most of the mechanisms of capacity-limited kinetics are probably related to the saturation of metabolic enzymes, a classic example being the metabolism of phenytoin (32). The pharmacokinetic data obtained from piglets disclosed that the kinetics was nonlinear and saturable if the plasma concentration of ponazuril was near or above K_m_ (10.8 μg/mL), and became linear at plasma concentrations below about 1 μg/mL. In clinical applications, the metabolic profile of ponazuril caused its slow elimination and significantly prolonged its residence time *in vivo*, as has been described in various animals. The average plasma elimination half-life (T_1/2_) of ponazuril in cattle has been reported to be around 58 h (23). However, plasma concentrations of ponazuril in the goats peaked at about 36 h after a single oral administration and were then eliminated with an average T_1/2_ of 129 h (24). In llamas, the serum concentration of ponazuril peaked at 84 h after a single dose of 20 mg/kg bw, with a reported elimination T_1/2_ of 135.5 h (25). After a single oral dose of ponazuril at 50 mg/kg in cats, the C_max_ in plasma was relatively low at 7.49 ug/ml with a T_max_ of 14.67 h, while the elimination T_1/2_ was 136 h. But the authors did not further describe possible non-linear absorption at such high administered doses (26). And in the study of Zou et al., the T_1/2_ of ponazuril in pigs after single oral administration was estimated as 135 h (29). Before the present work, few studies have evaluated the oral bioavailability of ponazuril using intravascular administration in these animals, and their pharmacokinetic profiles show varying degrees of interspecies variation. Since ponazuril shows Michaelis-Menten kinetics in some cases, its actual origins of metabolism and clearance need to be further elucidated.

According to the data of monitoring in feces and urine, the excretion of ponazuril in the feces rose rapidly from 0 to 60 h (2.5 d), cumulatively reaching more than 50% of the administered amounts, which should contain the unabsorbed portion of the gastrointestinal tract and the parent compound that was excreted *via* the systemic circulation, and then the excretion rate slowed down until it was no longer quantifiable at 972 h (40.5 d) after dosing, with the total amount of ponazuril in the feces reaching 86.42 ± 2.96%. Meanwhile, the excretion of ponazuril in the urine was less until a total amount of 0.31 ± 0.08% was recovered at 300 h (12.5 d) after dosing (Figure 8). These results suggest that ponazuril was excreted slowly from piglets after oral administration mainly in the prototype form through feces. A radiotracer study of ^14^C-toltrazuril in piglets following oral administration noted that the major route of excretion was *via* feces (73.33% of the recovery radioactivity), and the main metabolites in the feces were toltrazuril and toltrazuril sulfone (ponazuril), which accounted for 12.88 and 71.27% of the radioactivity at 21 days post-dose, respectively (6). Therefore, we previously inferred that most of the ponazuril in piglets remained to be excreted in parent form through feces, and the results also tentatively support this presumption.

Fortunately, ponazuril exhibits lower toxicity than its parent compound, toltrazuril, in rats and dogs with higher non-observed effect level (NOEL) (5). Toltrazuril was considered practically non-toxic with an oral LD_50_ of >5000 mg/kg in mice and was well tolerated by 7-day-old piglets at 100 mg/kg bw orally, which is 5 times the recommended dose (6). It suggested a wide margin of safety after oral administration of this series of drugs. As for therapeutic levels, although no pharmacodynamic studies *in vitro* have been reported to evaluate the therapeutic level of ponazuril against *C. suis*, Lindsay et al. demonstrated that 1 μg/mL ponazuril was effective in inhibiting the merozoite production over 90% of *Sarcocystis neurona* (47). And Mitchell et al. determined that ponazuril significantly inhibited *Toxoplasma gondii* tachyzoite production at 0.1–5 μg/mL in African green monkey kidney cells (48). Corresponding to nonlinear pharmacokinetic observations in piglets, the blood concentration of ponazuril remained above 1 ug/mL until approximately 480 h after a single oral dose of 20 mg/kg bw, which is essential for the potential therapeutic concentration. In addition, Karembe et al. reported the disposition kinetics of toltrazuril and ponazuril in plasma and the intestinal tissues after oral and intramuscular application of toltrazuril in piglets. This study observed that after the metabolism of toltrazuril to ponazuril, both showed significant and sustained concentrations in the jejunal tissue where *C. suis* mainly colonize, as well as in the intestinal contents (49). We have not yet obtained sufficient data to demonstrate the mechanisms involved in the higher and more sustained plasma, tissue, and fecal concentrations. Ultimately, however, the maintenance of effective drug concentrations *in vivo* is beneficial in helping piglets against coccidiosis infection during the growth phase (50).

## Conclusion

This study first showed the absolute oral bioavailability of ponazuril in 2-week-old piglets with Michaelis-Menten elimination, and slow excretion largely *via* feces. The relevant pharmacokinetic parameters and excretion patterns obtained can also serve as a theoretical foundation for subsequent metabolic and tissue distribution studies of ponazuril, and provide a basis for its further progress toward application in food animals.

## Data availability statement

The original contributions presented in the study are included in the article/Supplementary material, further inquiries can be directed to the corresponding author.

## Ethics statement

The animal study was reviewed and approved by Animal Ethics Committee of South China Agriculture University.

## Author contributions

XH, LL, and HD: conceptualization, supervision, and project administration. WW, DH, WY, and XC: animal experiments. YM, JN, YZ, and YS: Sample processing and data analysis. YZ and JN: writing-original draft preparation. WW, LL, and YM: writing–review, editing, and revision. All authors contributed to manuscript revision, read, and approved the submitted version.

## References

[1] Joachim A Shrestha A. Coccidiosis of Pigs. Coccidiosis in Livestock, Poultry, Companion Animals, and Humans. Boca Raton, FL: CRC Press. (2019).

[2] Meyer C Joachim A Daugschies A. Occurrence of Isospora suis in larger piglet production units and on specialized piglet rearing farms. Vet Parasitol. (1999) 82:277–84. 10.1016/S0304-4017(99)00027-8 10384903

[3] Worliczek HL Buggelsheim M Saalmüller A Joachim A. Porcine isosporosis: Infection dynamics, pathophysiology and immunology of experimental infections. Wien Klin Wochenschr. (2007) 119:33–9. 10.1007/s00508-007-0859-3 17987356

[4] Noack S Chapman HD Selzer PM. Anticoccidial drugs of the livestock industry. Parasitol Res. (2019) 118:2009–26. 10.1007/s00436-019-06343-5 31152233 PMC6611755

[5] EMA. Committee for Veterinary Medicinal Products. Toltrazuril, Summary Report (1). (1998). Available online at: https://www.ema.europa.eu/en/documents/mrl-report/toltrazuril-summary-report-1-committee-veterinary-medicinal-products_en.pdf (accessed September 05, 2022).

[6] EMA. Committee for Veterinary Medicinal Products. Toltrazuril (Extension to Pigs), Summary Report (3). (2000). Available online at: https://www.ema.europa.eu/en/documents/mrl-report/toltrazuril-extension-pigs-summary-report-3-committee-veterinary-medicinal-products_en.pdf (accessed September 05, 2022).

[7] EMA. Committee for Veterinary Medicinal Products. Toltrazuril (Extension to Pigs), Summary Report (2). (1999). Available online at: https://www.ema.europa.eu/en/documents/mrl-report/toltrazuril-extension-pigs-summary-report-2-committee-veterinary-medicinal-products_en.pdf (accessed September 05, 2022).

[8] Stock ML Elazab ST Hsu WH. Review of triazine antiprotozoal drugs used in veterinary medicine. J Vet Pharmacol Ther. (2018) 41:184–94. 10.1111/jvp.12450 28833212

[9] Kandeel M. Efficacy of amprolium and toltrazuril in chicken with subclinical infection of cecal coccidiosis. Indian J Pharmacol. (2011) 43:741–3. 10.4103/0253-7613.89845 22144793 PMC3229804

[10] Harder A Haberkorn A. Possible mode of action of toltrazuril: studies on two Eimeria species and mammalian and Ascaris suum enzymes. Parasitol Res. (1989) 76:8–12. 10.1007/BF00931064 2560189

[11] Joachim A Altreuther G Bangoura B Charles S Daugschies A Hinney B et al. W A A V P Guideline for Evaluating the Efficacy of Anticoccidials in Mammals (Pigs, Dogs, Cattle, Sheep). Vet Parasitol. (2018) 253:102–19. 10.1016/j.vetpar.2018.02.029 29604993

[12] Joachim A Shrestha A Freudenschuss B Palmieri N Hinney B Karembe H et al. Comparison of an injectable toltrazuril-gleptoferron (Forceris^®^) and an oral toltrazuril (Baycox^®^) + injectable iron dextran for the control of experimentally induced piglet cystoisosporosis. Parasite Vector. (2018) 11:206. 10.1186/s13071-018-2797-5 29580269 PMC5870915

[13] Zapa DMB Couto LFM Heller LM Ferreira LL Iuasse HV Naves RB. Long-term efficacy of toltrazuril in nave calves prophylactically treated and experimentally infected with Eimeria spp. Parasitol Res. (2022) 121:2571–8. 10.1007/s00436-022-07601-9 35895113

[14] NCBI. National Center for Biotechnology Information. PubChem Compound Summary for CID 3050408, Ponazuril. (2022). Available online at: https://pubchem.ncbi.nlm.nih.gov/compound/Ponazuril (accessed September 05, 2022).

[15] FDA. Freedom of information summary. Original New Animal Drug Application. Marquis™ *(15% w/w ponazuril) Antiprotozoal Oral Paste*. (2001). Available online at: https://animaldrugsatfda.fda.gov/adafda/app/search/public/document/downloadFoi/698 (accessed September 05, 2022).

[16] Dubey JP Lindsay DS Saville WJA Reed SM Granstrom DE Speer CA. review of Sarcocystis neurona and equine protozoal myeloencephalitis (EPM). Vet Parasitol. (2001) 95:89–131. 10.1016/S0304-4017(00)00384-8 11223193

[17] Mitchell SM Zajac AM Davis WL Kennedy TJ Lindsay DS. The effects of ponazuril on development of apicomplexans in vitro. J Eukaryot Microbiol. (2005) 52:231–5. 10.1111/j.1550-7408.2005.00029.x 15926999

[18] Qiu X. Study of Pharmacodynamics and Safety of Pharmacology of Toltrazuril Sulfone in Swine Coccidiosis. Nanchang, China: Jiangxi Agricultural University. (2013).

[19] Wise LN Ueti MW Kappmeyer LS Hines MT White SN Davis W et al. *In vitro* activity of ponazuril against Theileria equi. Vet Parasitol. (2012) 185:282–5. 10.1016/j.vetpar.2011.10.036 22130334

[20] Lim JH Kim MS Hwang YH Song IB Park BK Yun HI. Pharmacokinetics of toltrazuril and its metabolites, toltrazuril sulfoxide and toltrazuril sulfone, after a single oral administration to pigs. J Vet Med Sci. (2010) 72:1085–7. 10.1292/jvms.09-0524 20332592

[21] Atef M El-Banna HA Elzorba HY Soliman AM. Pharmacokinetics and tissue residue of enrofloxacin in healthy, Eimeria-infected broiler chickens and those pre-treated with amprolium and toltrazuril. Int J Vet Sci Med. (2020) 8:31–8. 10.1080/23144599.2020.1765720 32923475 PMC7448909

[22] Kim MS Lim JH Hwang YH Park BK Yun HI. Plasma disposition of toltrazuril and its metabolites, toltrazuril sulfoxide and toltrazuril sulfone, in rabbits after oral administration. Vet Parasitol. (2010) 169:51–6. 10.1016/j.vetpar.2009.12.011 20083354

[23] Dirikolu L Yohn R Garrett EF Chakkath T Ferguson DC. Detection, quantifications and pharmacokinetics of toltrazuril sulfone (Ponazuril) in cattle. J Vet Pharmacol Ther. (2009) 32:280–8. 10.1111/j.1365-2885.2008.01039.x 19646093

[24] Love D Gibbons P Fajt V Jones M. Pharmacokinetics of single-dose oral ponazuril in weanling goats. J Vet Pharmacol Ther. (2016) 39:305–8. 10.1111/jvp.12273 26542450

[25] Prado ME Ryman JT Boileau MJ Martin-Jimenez T Meibohm B. Pharmacokinetics of ponazuril after oral administration to healthy llamas (Lama glama). Am J Vet Res. (2011) 72:1386–9. 10.2460/ajvr.72.10.1386 21962282

[26] Burlison C Cox S Smith J Stokes J Whittemore JC DeBolt B. Pharmacokinetics of orally administered single-dose ponazuril in cats. J Vet Pharmacol Ther. (2022) 45:229–34. 10.1111/jvp.13047 35307837 PMC9311793

[27] Furr M Kennedy T. Effects of coadministration of corn oil and ponazuril on serum and cerebrospinal fluid concentrations of ponazuril in horses. J Vet Intern Med. (2020) 34:1321–4. 10.1111/jvim.15765 32301131 PMC7255669

[28] Jacobson ER Stacy NI Mader DR Moretti R Zirkelbach B Carlile O. Pharmacokinetics of ponazuril after administration of a single oral dose to green turtles (Chelonia mydas). Vet Quart. (2021) 41:323–31. 10.1080/01652176.2021.2008045 34789079 PMC8667943

[29] Zou M Guo G Zhao Y Zhang Q. Detection, quantifications, and pharmacokinetics of ponazuril in healthy swine. J Vet Pharmacol Ther. (2014) 37:598–602. 10.1111/jvp.12126 24731142

[30] Ministry of Agriculture Rural Affairs P. Announcement No. 216. (2019). Available online at: https://www.moa.gov.cn/gk/tzgg_1/gg/201909/t20190923_6328783.htm (accessed September 05, 2022).

[31] Rubin GM Tozer TN. Theoretical considerations in the calculation of bioavailability of drugs exhibiting Michaelis-Menten elimination kinetics. J Pharmacokinet Biopharm. (1984) 12:437–50. 10.1007/BF01062667 6527233

[32] Jusko WJ Koup JR Alvan G. Nonlinear assessment of phenytoin bioavailability. J Pharmacokinet Biopharm. (1976) 4:327–36. 10.1007/BF01063122 978395

[33] Mroz Z Jongbloed AW Lenis NP Vreman K. Water in pig nutrition: physiology, allowances and environmental implications. Nutr Res Rev. (1995) 8:137–64. 10.1079/NRR19950010 19094283

[34] Tollerz G. Volume of distribution of tritiated water as a measure of total body water in suckling pigs. Acta Vet Scand. (1964) 5:24–34. 10.1186/BF03547362

[35] Shargel L Yu ABC. Applied Biopharmaceutics & Pharmacokinetics. New York, NY: McGraw-Hill Education. (2016).

[36] Tozer Mrtn. Clinical pharmacokinetics and pharmacodynamics concepts and applications. Philadelphia/Baltimore: Wolters Kluwer Health/Lippincott William & Wilkins. (2011).

[37] Zec SH Papich MG Oehler DA Hills K Schmid S Huth K. Pharmacokinetics of a single oral dose of ponazuril in the indian peafowl (Pavo cristatus). J Zoo Wildl Med. (2021) 52:548–54. 10.1638/2020-0026 34130397

[38] Zabielski R Le Huerou-Luron I Guilloteau P. Development of gastrointestinal and pancreatic functions in mammalians (mainly bovine and porcine species): influence of age and ingested food. Reprod Nutr Dev. (1999) 39:5–26. 10.1051/rnd:19990101 10222497

[39] Makkink CA Negulescu GP Qin G Verstegen MW. Effect of dietary protein source on feed intake, growth, pancreatic enzyme activities and jejunal morphology in newly-weaned piglets. Br J Nutr. (1994) 72:353–68. 10.1079/BJN19940039 7947652

[40] Wang J Liu B Shi Y. Pig Growth and Nutrition Control Technology. Beijing: China Agricultural University Press. (2014).

[41] Snoeck V Huyghebaert N Cox E Vermeire A Saunders J Remon JP et al. Gastrointestinal transit time of nondisintegrating radio-opaque pellets in suckling and recently weaned piglets. J Control Release. (2004) 94:143–53. 10.1016/j.jconrel.2003.09.015 14684278

[42] Gregory PC McFadyen M Rayner DV. Pattern of gastric emptying in the pig: relation to feeding. Br J Nutr. (1990) 64:45–58. 10.1079/BJN19900008 2400768

[43] Clemens ET Stevens CE Southworth M. Sites of organic acid production and pattern of digesta movement in the gastrointestinal tract of swine. J Nutr. (1975) 105:759–68. 10.1093/jn/105.6.759 237989

[44] Ludden TM. Nonlinear pharmacokinetics: clinical Implications. Clin Pharmacokinet. (1991) 20:429–46. 10.2165/00003088-199120060-00001 2044328

[45] Li B Wang Y Feng Y Yuan D Xu R Jiang C et al. Design and molecular insights of drug-active metabolite based co-amorphous formulation: a case study of toltrazuril-ponazuril co-amorphous. Int J Pharmaceut. (2022) 615:121475. 10.1016/j.ijpharm.2022.121475 35041914

[46] Dirikolu L Karpiesiuk W Lehner AF Hughes C Granstrom DE Tobin T. Synthesis and detection of toltrazuril sulfone and its pharmacokinetics in horses following administration in dimethylsulfoxide. J Vet Pharmacol Ther. (2009) 32:368–78. 10.1111/j.1365-2885.2008.01053.x 19614842

[47] Lindsay DS Dubey JP Kennedy TJ. Determination of the activity of ponazuril against Sarcocystis neurona in cell cultures. Vet Parasitol. (2000) 92:165–9. 10.1016/S0304-4017(00)00280-6 10946140

[48] Mitchell SM Zajac AM Davis WL Lindsay DS. Efficacy of ponazuril in vitro and in preventing and treating Toxoplasma gondii infections in mice. J Parasitol. (2004) 90:639–42. 10.1645/GE-250R 15270113

[49] Karembe H Sperling D Varinot N Magnier R Peyrou M Guerra N. Absorption and distribution of toltrazuril and toltrazuril sulfone in plasma, intestinal tissues and content of piglets after oral or intramuscular administration. Molecules. (2021) 26:5633. 10.3390/molecules26185633 34577103 PMC8468611

[50] Hiob L Holzhausen I Sperling D Pagny G Meppiel L Isaka N. Efficacy of an injectable toltrazuril–gleptoferron (Forceris^®^) to control coccidiosis (Cystoisospora suis) in comparison with iron supplemented piglets without anticoccidial treatment. Vet Parasitol X. (2019) 1:100002. 10.1016/j.vpoa.2019.100002 32904741 PMC7458377

