# Supplementary material for: Pharmacokinetics, bioavailability, and excretion of ponazuril in piglets
Source: Front Vet Sci. 2022 Dec 7;9:1054417. doi: 10.3389/fvets.2022.1054417 (PMC9768325; doi:10.3389/fvets.2022.1054417)
Supplement: Supplementary file 1 [file Data_Sheet_1.docx]

Supplementary Material

# Supplementary Table

**Supplementary Table 1.** Plasma concentration of ponazuril in piglets after intravenous administration of 20 mg/kg bw

| **Time (h)** | **Plasma concentration of piglet No. 1-6 (μg/mL)** | | | | | |
| --- | --- | --- | --- | --- | --- | --- |
|  | **1** | **2** | **3** | **4** | **5** | **6** |
| 0.17 | 47.64 | 43.13 | 39.82 | 41.41 | 32.38 | 30.93 |
| 1 | 42.48 | 38.71 | 34.69 | 40.73 | 26.40 | 29.34 |
| 6 | 41.05 | 35.74 | 33.09 | 35.51 | 25.60 | 29.07 |
| 12 | 40.12 | 35.47 | 29.90 | 35.12 | 25.46 | 28.93 |
| 24 | 39.92 | 32.62 | 29.69 | 32.21 | 24.39 | 28.83 |
| 48 | 39.05 | 32.39 | 26.03 | 30.36 | 24.31 | 28.12 |
| 72 | 34.17 | 30.93 | 20.17 | 30.17 | 22.95 | 25.45 |
| 96 | 29.15 | 30.63 | 19.32 | 28.92 | 22.96 | 24.02 |
| 144 | 21.37 | 20.76 | 18.74 | 22.85 | 21.67 | 23.69 |
| 192 | 15.80 | 14.77 | 17.90 | 17.07 | 16.74 | 18.88 |
| 240 | 12.05 | 8.93 | 10.66 | 10.98 | 11.32 | 15.73 |
| 360 | 3.27 | 2.92 | 3.51 | 2.27 | 2.95 | 5.45 |
| 480 | 1.03 | 1.34 | 1.07 | 0.92 | 0.97 | 1.37 |
| 600 | 0.50 | 0.38 | 0.35 | 0.35 | 0.36 | 0.39 |
| 720 | 0.21 | 0.12 | 0.18 | 0.21 | 0.10 | 0.15 |
| 840 | 0.17 | <LOQ | <LOQ | 0.10 | <LOQ | <LOQ |
| 960 | <LOQ | <LOQ | <LOQ | <LOQ | <LOQ | <LOQ |

<LOQ, concentration below the limit of quantification

**Supplementary Table 2.** Plasma concentration of ponazuril in piglets after oral administration of 20 mg/kg bw

| **Time (h)** | **Plasma concentration of piglet No. 7-12 (μg/mL)** | | | | | |
| --- | --- | --- | --- | --- | --- | --- |
|  | **7** | **8** | **9** | **10** | **11** | **12** |
| 0.5 | 0.31 | 0.52 | 0.15 | 0.30 | 0.23 | 0.30 |
| 1 | 1.07 | 0.84 | 0.66 | 1.45 | 0.68 | 0.81 |
| 6 | 4.60 | 3.77 | 4.17 | 7.50 | 6.09 | 7.99 |
| 12 | 9.32 | 7.35 | 8.57 | 11.58 | 13.44 | 11.95 |
| 24 | 10.40 | 11.67 | 12.36 | 14.18 | 17.91 | 14.04 |
| 36 | 15.79 | 18.09 | 18.43 | 16.51 | 17.92 | 17.31 |
| 48 | 17.44 | 19.67 | 17.77 | 17.77 | 19.95 | 16.31 |
| 72 | 15.48 | 19.99 | 15.96 | 14.65 | 19.78 | 15.60 |
| 96 | 12.95 | 17.97 | 15.85 | 14.32 | 18.19 | 13.78 |
| 144 | 12.92 | 16.39 | 12.11 | 11.47 | 13.45 | 12.79 |
| 240 | 8.67 | 7.13 | 8.30 | 5.68 | 6.29 | 7.48 |
| 360 | 3.49 | 2.63 | 4.65 | 2.11 | 2.24 | 2.82 |
| 480 | 1.08 | 0.83 | 1.80 | 0.97 | 1.03 | 1.07 |
| 600 | 0.66 | 0.44 | 0.74 | 0.52 | 0.54 | 0.46 |
| 720 | 0.19 | 0.25 | 0.34 | 0.24 | 0.26 | 0.19 |
| 840 | <LOQ | 0.10 | 0.19 | 0.11 | 0.13 | 0.12 |
| 960 | <LOQ | <LOQ | <LOQ | <LOQ | <LOQ | <LOQ |

**Supplementary Table 3a.** Mean excretion of ponazuril in fecal samples at various times ($\bar{X}$±SD, n=6)

| **Time (h)** | **Average excretion amounts (mg)** | **Percentage of administered dose (%)** | **Percentage of cumulative excretion (%)** | **Cumulative percentage of dosing (%)** |
| --- | --- | --- | --- | --- |
| 0-12 | 4.02±2.38 | 7.66±5.23 | 8.84±5.95 | 7.66±5.23 |
| 12-24 | 7.69±1.83 | 14.43±4.26 | 16.60±4.62 | 22.09±8.44 |
| 24-36 | 10.85±3.69 | 19.81±6.25 | 22.89±6.97 | 41.90±7.14 |
| 36-60 | 5.55±2.80 | 10.04±4.83 | 11.63±5.55 | 51.94±7.24 |
| 60-84 | 2.89±1.54 | 5.15±2.39 | 6.00±2.91 | 57.10±7.63 |
| 84-108 | 1.74±0.67 | 3.23±1.35 | 3.72±1.49 | 60.33±8.39 |
| 108-132 | 1.35±0.53 | 2.47±0.93 | 2.86±1.09 | 62.80±8.88 |
| 132-156 | 1.47±0.56 | 2.69±0.94 | 3.13±1.13 | 65.49±8.57 |
| 156-180 | 1.19±0.48 | 2.17±0.77 | 2.52±0.95 | 67.65±7.91 |
| 180-204 | 1.08±0.58 | 1.98±1.08 | 2.30±1.26 | 69.63±7.13 |
| 204-228 | 0.89±0.82 | 1.58±1.39 | 1.86±1.68 | 71.21±6.17 |
| 228-252 | 1.05±0.35 | 1.94±0.66 | 2.25±0.78 | 73.15±5.92 |
| 252-276 | 0.64±0.49 | 1.19±0.95 | 1.38±1.09 | 74.34±5.37 |
| 276-300 | 0.60±0.35 | 1.09±0.66 | 1.27±0.75 | 75.43±5.43 |
| 300-324 | 0.50±0.34 | 0.89±0.53 | 1.05±0.65 | 76.33±5.19 |
| 324-348 | 0.65±0.58 | 1.21±1.13 | 1.41±1.31 | 77.54±4.66 |
| 348-372 | 0.53±0.26 | 1.00±0.58 | 1.16±0.66 | 78.54±4.40 |
| 372-396 | 0.44±0.21 | 0.81±0.33 | 0.94±0.41 | 79.34±4.17 |
| 396-420 | 0.51±0.31 | 0.92±0.53 | 1.08±0.63 | 80.27±3.77 |
| 420-444 | 0.25±0.16 | 0.46±0.31 | 0.53±0.36 | 80.72±3.68 |
| 444-468 | 0.43±0.25 | 0.81±0.50 | 0.93±0.57 | 81.53±3.53 |
| 468-492 | 0.35±0.18 | 0.66±0.39 | 0.76±0.44 | 82.19±3.26 |

**Supplementary Table 3b.** Continuation of Supplementary table 3a

| **Time (h)** | **Average excretion amounts (mg)** | **Percentage of administered dose (%)** | **Percentage of cumulative excretion (%)** | **Cumulative percentage of dosing (%)** |
| --- | --- | --- | --- | --- |
| 492-516 | 0.32±0.13 | 0.60±0.26 | 0.69±0.31 | 82.79±3.13 |
| 516-540 | 0.31±0.18 | 0.58±0.38 | 0.67±0.43 | 83.36±2.86 |
| 540-564 | 0.23±0.21 | 0.44±0.44 | 0.50±0.50 | 83.80±2.67 |
| 564-588 | 0.20±0.11 | 0.38±0.22 | 0.44±0.25 | 84.18±2.67 |
| 588-612 | 0.16±0.14 | 0.29±0.27 | 0.34±0.31 | 84.47±2.68 |
| 612-636 | 0.23±0.22 | 0.44±0.46 | 0.50±0.52 | 84.91±2.67 |
| 636-660 | 0.11±0.10 | 0.21±0.22 | 0.24±0.25 | 85.12±2.67 |
| 660-684 | 0.10±0.06 | 0.18±0.11 | 0.21±0.12 | 85.30±2.67 |
| 684-708 | 0.06±0.02 | 0.11±0.05 | 0.13±0.06 | 85.41±2.67 |
| 708-732 | 0.10±0.07 | 0.20±0.15 | 0.22±0.17 | 85.61±2.75 |
| 732-756 | 0.07±0.05 | 0.12±0.11 | 0.14±0.12 | 85.73±2.77 |
| 756-780 | 0.12±0.09 | 0.22±0.18 | 0.25±0.20 | 85.95±2.80 |
| 780-804 | 0.08±0.07 | 0.16±0.13 | 0.18±0.15 | 86.11±2.85 |
| 804-828 | 0.05±0.02 | 0.08±0.05 | 0.10±0.06 | 86.19±2.86 |
| 828-852 | 0.04±0.02 | 0.08±0.04 | 0.09±0.05 | 86.27±2.87 |
| 852-876 | 0.02±0.01 | 0.04±0.03 | 0.05±0.03 | 86.31±2.89 |
| 876-900 | 0.02±0.02 | 0.04±0.04 | 0.04±0.05 | 86.35±2.91 |
| 900-924 | 0.02±0.02 | 0.04±0.04 | 0.04±0.05 | 86.38±2.93 |
| 924-948 | 0.01±0.02 | 0.03±0.03 | 0.03±0.04 | 86.41±2.95 |
| 948-972 | 0.01±0.01 | 0.01±0.02 | 0.01±0.03 | 86.42±2.96 |
| 972-996 | ND | ND | ND | 86.42±2.96 |
| 996-1020 | ND | ND | ND |  |

ND: not detected.

**Supplementary Table 4.** Mean excretion of ponazuril in urine samples at various times

($\bar{X}$±SD, n=6)

| **Time (h)** | **Average excretion amounts (mg)** | **Percentage of administered dose (%)** | **Percentage of cumulative excretion (%)** | **Cumulative percentage of dosing (%)** |
| --- | --- | --- | --- | --- |
| 0-12 | 0.031±0.019 | 0.059±0.038 | 17.85±9.97 | 0.06±0.04 |
| 12-24 | 0.032±0.016 | 0.057±0.026 | 20.19±12.13 | 0.12±0.04 |
| 24-36 | 0.022±0.009 | 0.042±0.021 | 13.24±4.57 | 0.16±0.04 |
| 36-60 | 0.010±0.004 | 0.020±0.008 | 6.25±2.01 | 0.18±0.05 |
| 60-84 | 0.012±0.008 | 0.022±0.015 | 7.05±4.00 | 0.20±0.06 |
| 84-108 | 0.009±0.005 | 0.016±0.010 | 4.91±2.22 | 0.22±0.07 |
| 108-132 | 0.006±0.002 | 0.012±0.005 | 3.69±1.34 | 0.23±0.07 |
| 132-156 | 0.012±0.007 | 0.023±0.014 | 7.30±3.19 | 0.25±0.08 |
| 156-180 | 0.008±0.004 | 0.014±0.009 | 4.65±2.90 | 0.26±0.08 |
| 180-204 | 0.006±0.004 | 0.012±0.008 | 3.85±2.43 | 0.28±0.08 |
| 204-228 | 0.011±0.012 | 0.019±0.018 | 6.80±7.91 | 0.30±0.07 |
| 228-252 | 0.004±0.004 | 0.008±0.009 | 2.30±2.26 | 0.30±0.08 |
| 252-276 | 0.002±0.002 | 0.004±0.005 | 1.16±1.41 | 0.31±0.08 |
| 276-300 | 0.001±0.002 | 0.003±0.004 | 0.75±1.19 | 0.31±0.08 |
| 300-324 | ND | ND | ND | 0.31±0.08 |
| 324-1020 | ND | ND | ND |  |
